# Integrated identification of growth pattern and taxon of bacterium in gut microbiota via confocal fluorescence imaging‐oriented single‐cell sequencing

**DOI:** 10.1002/mlf2.12041

**Published:** 2022-09-26

**Authors:** Juan Gao, Di Sun, Bei Li, Chaoyong Yang, Wei Wang

**Affiliations:** ^1^ Shanghai Key Laboratory for Nucleic Acid Chemistry and Nanomedicine, Institute of Molecular Medicine, Renji Hospital Shanghai Jiao Tong University School of Medicine Shanghai China; ^2^ State Key Laboratory of Applied Optics, Changchun Institute of Optics, Fine Mechanics and Physics Chinese Academy of Sciences Changchun China; ^3^ The MOE Key Laboratory of Spectrochemical Analysis and Instrumentation, Key Laboratory for Chemical Biology of Fujian Province State Key Laboratory of Physical Chemistry of Solid Surfaces, Department of Chemical Biology, Xiamen University College of Chemistry and Chemical Engineering Xiamen China; ^4^ University of Oxford UK

**Keywords:** bacterial growth pattern, confocal imaging, fluorescent d‐amino acid probe, in vivo labeling, single‐cell sequencing

## Abstract

Despite the fast progress in our understanding of the complex functions of gut microbiota, it is still challenging to directly investigate the in vivo microbial activities and processes on an individual cell basis. To gain knowledge of the indigenous growth/division patterns of the diverse mouse gut bacteria with a relatively high throughput, here, we propose an integrative strategy, which combines the use of fluorescent probe labeling, confocal imaging with single‐cell sorting, and sequencing. Mouse gut bacteria sequentially labeled by two fluorescent d‐amino acid probes in vivo were first imaged by confocal microscopy to visualize their growth patterns, which can be unveiled by the distribution of the two fluorescence signals on each bacterium. Bacterial cells of interest on the imaging slide were then sorted using a laser ejection equipment, and the collected cells were then sequenced individually to identify their taxa. Our strategy allows integrated acquirement of the growth pattern knowledge of a variety of gut bacteria and their genomic information on a single‐cell basis, which should also have great potential in studying many other complex bacterial systems.

## Impact statement

Bacteria residing in the mammalian gut have diverse cellular morphologies, but our knowledge of this topic is still very limited. Previously, we developed an in vivo labeling protocol where fluorescent d‐amino acid probes were sequentially given to mouse via gavage to record the in situ growing processes of its gut microbes. However, identifying the taxa of a particular bacterium that showed interesting growth or division modes was still very challenging. Here, we integrate the use of confocal fluorescence imaging with single‐cell sorting and sequencing, which allows directly isolating the cell of interest under a confocal microscope and then identifying its taxa.

## INTRODUCTION

The highly diverse bacteria in the mammalian gut play multifaceted roles in influencing the host metabolism, immunity, and neurobehavioral functions^1^, ^2^. Understanding the in vivo microbial processes and activities of different gut bacteria is instrumental in comprehending these functions and their interactions with the host. Unfortunately, a majority of gut bacteria in both human and model animals like mouse and rat have not yet been obtained in pure culture^2^. As a result, researchers often have to rely on molecular methods, like 16S ribosomal DNA (rDNA) and metagenomic sequencing, to investigate this gut “dark matter”^3^. The sequencing‐based strategies, however, can only provide limited knowledge of the phenotypes of gut bacteria, which hinders our exploration of the microbial activities and functions, especially for those that occur in vivo.

As a complement to traditional bulk sequencing, single‐cell sequencing has been applied in deciphering the genomes of different bacteria collected from a variety of environments in recent years, which also included a high number of microbes that are unculturable in vitro^4^, ^5^, ^6^, ^7^. Since the implementation of this technique, it has been very desirable to couple the phenotypic with genotypic information on a single‐cell basis. Being able to observe and sort the bacterial cells of interest and directly identify their taxa, or even ascertain the genome, at a relatively high throughput, will be very valuable to the microbiology field. However, due to the technical challenges in simultaneously obtaining phenotypic data and manipulating a single bacterium, it has been highly difficult to put this schema into practice.

A recent successful use of this approach in microbiota research combined the employment of Raman‐based single‐cell analysis together with cell sorting and sequencing^8^, ^9^, ^10^, ^11^, ^12^. In this strategy, heavy water (D_2_O) was used to label the metabolically active cells in the gut microbiotas, which were cultured in vitro. The accumulated incorporation of deuterium of single cells could then be analyzed by Raman micro‐spectroscopy, and the bacteria of interest were then sorted via optical tweezer or laser ejection and subjected to single‐cell sequencing (dozens of cells were combined for sequencing in the earlier work^8^). This technique enabled linking the metabolic status and genomic information of bacterium on a single‐cell basis to some extent. However, due to the requirement of high concentrations of D_2_O in labeling, the uses of this technique have been limited to microbiotas cultured in vitro^5^, ^13^.

Compared with Raman‐based bacterial analysis, fluorescence imaging, which is often considered as the working horse in modern biology research, has several inherent advantages, including its ease of use, the abundant fluorescent labeling tags and probes to choose from, and more importantly, the detailed phenotypic information obtainable through advanced fluorescence imaging. Recently, our group developed an in vivo fluorescence labeling strategy for revealing the indigenous growth processes and division patterns of gut bacteria in the mammalian hosts^14^. In the protocol, fluorescent d‐amino acid (FDAA) probes, which could label the bacterial peptidoglycan via the functioning of their l,d‐ and d,d‐transpeptidases^15^, ^16^, were sequentially given to mouse via gavage. This resulted in the two‐color labeling of a majority of the gut bacteria, and the distributions of the two signals on each bacterium contained clues of their cell wall synthesis during the period of the labeling (the more actively constructing sites would have stronger labeling of the second FDAA). Next, to gain taxonomic information on the labeled bacteria, we resorted to fluorescence in situ hybridization (FISH)^14^, ^17^. Parallel FISH staining with many DNA probes targeting specific bacterial taxa within the sample was performed. The FISH signals, together with the FDAA‐labeling signals, could thus unveil the growth patterns of the corresponding bacterial taxa. Nonetheless, it is well known that designing new FISH probes against a particular taxon with high specificity is often very challenging, partially due to the presence of a large number of unidentified bacteria in the gut microbiota^14^. In addition, optimizing and carrying out the FISH protocol is labor intensive and time consuming. These issues prevented the identification of bacterial growth patterns in the gut microbiota with a high throughput. Therefore, we asked whether it is possible to directly identify the taxa of the bacteria having interesting fluorescence labeling under a confocal microscope.

**Figure 1:**
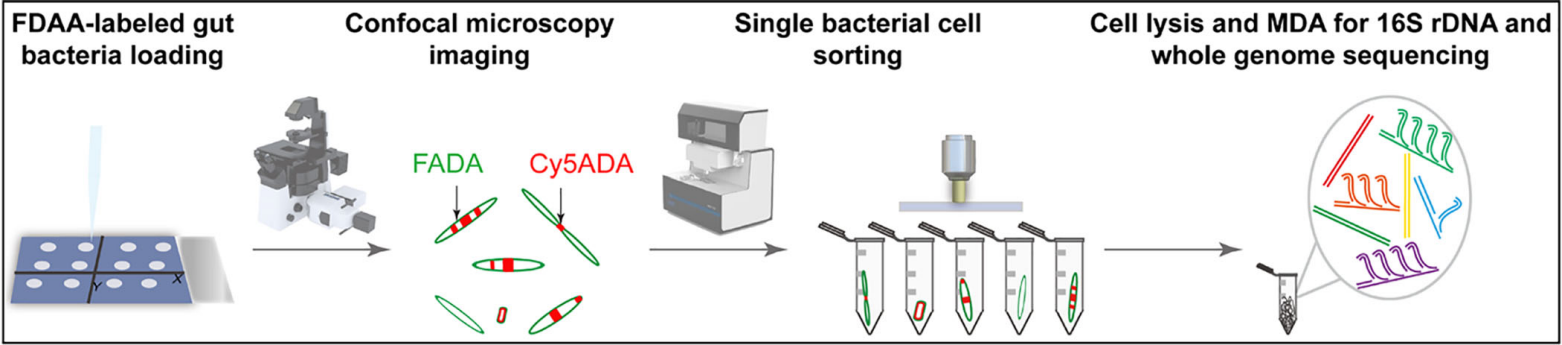
Schematic overview of FLCiSS. Sequentially labeled cecal microbiota by FDAAs was collected and loaded onto a glass slide with a coordinate axis designed for locating targeted cells during imaging and sorting. Morphology and growth/division patterns of the bacteria were directly imaged by confocal fluorescence microscopy without a cover slide. The coordinates of the bacteria of interest were recorded. The glass slide was then transferred to a cell‐ejection equipment and targeted cells were sorted into sterile tubes individually via laser ejection. After cell lysis, the 16S rRNA gene of the bacterium was then amplified via MDA before sequencing for taxonomic identification; the cells yielding positive 16S rRNA gene amplicons were further used for library construction and whole‐genome sequencing. FDAA, fluorescent d‐amino acid; FLCiSS, fluorescent probe labeling and confocal imaging with single‐cell sorting and sequencing; MDA, multiple displacement amplification; rRNA, ribosomal RNA.

Herein, we propose an integrative strategy, combining fluorescent probe labeling and confocal imaging with single‐cell sorting and sequencing (FLCiSS, workflow shown in Figure 1), which allows integrated acquirement of the growth/division pattern and the genomic knowledge of gut bacteria on a single‐cell basis. In this strategy, fluorescently labeled bacteria are first loaded onto a sorting chip that has a coordinate axis for locating the cells and a layer of special cladding material for the following laser ejection. After air‐drying, the samples are directly analyzed (without a cover slide) with a confocal fluorescence microscope to visualize their labeling signals. The coordinates of the bacteria with interesting labeling patterns are recorded, and the chip is then transferred to a single‐cell ejection equipment (PRECI SCS; HOOKE Instruments) to visually collect the recorded cell of interest (located based on their coordinates) into sterile receiving tubes by a laser beam. After lysis of the sorted cells, multiple displacement amplification (MDA), 16S rDNA and whole‐genome sequencing are then performed, thus enabling a direct link between the growth pattern phenotype and the taxonomic identity of the individual bacterium.

## RESULTS AND DISCUSSION

### Assessing the practicability of FLCiSS with a mock bacterial community

**Figure 2:**
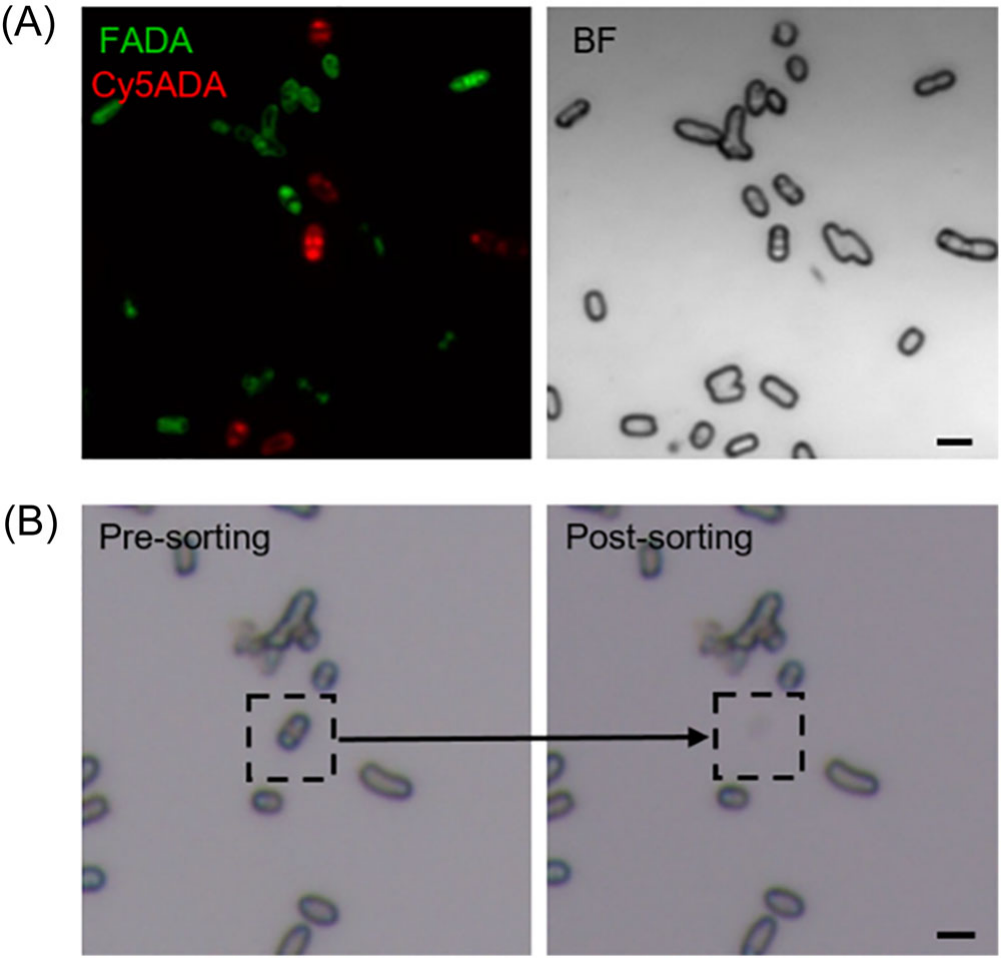
Analysis of a mock bacterial community by FLCiSS. (A) Confocal imaging of the mock sample. *Escherichia coli* and *Bacillus subtilis* separately prelabeled with FADA (green) and Cy5ADA (red) were mixed and imaged after loading on the special slide. Scale bar = 2 μm. (B) An example of the BF images before and after a bacterium (boxed) was collected, indicating the cell was successfully sorted. Scale bar = 2 μm. BF, bright field.

To determine the practicability of FLCiSS, we first tested the protocol with an artificial bacterial community, which contained a strain of *Escherichia coli* (K12) and a strain of *Bacillus subtilis* (CICC 23659) separately prelabeled with carboxyfluorescein‐amino‐d‐alanine (FADA) and Cy5‐amino‐d‐alanine (Cy5ADA). The mixed bacterial sample (10 μl) was loaded onto a sorting chip (HSC24 chip; HOOKE Instruments) for confocal imaging (S3000; HOOKE Instruments), and the two species could be well‐differentiated via their labeling signals (Figure 2A). Based on their fluorescence, 30 cells of each strain were then sorted into separate receiving tubes (HSR04; HOOKE Instruments). Representative bright field images of the sample before and after sorting of a bacterial cell are shown in Figure 2B. A notorious technical challenge in single bacterium sequencing is the rigid cell wall in Gram‐positive bacteria, which makes the isolation of their genome very difficult^18^. To improve the cell lysis efficiency, we optimized several steps in the protocol, including the pretreatment step (freeze–thaw cycles), lysis buffer, and incubation time (see Materials and methods section for details). After cell lysis, genomic DNA (gDNA) was amplified by MDA^19^, and concentrations of gDNA ranging from 200 to 700 ng/μl with a total volume of ~10 μl were attained, indicating an effective DNA extraction of both species. Used as negative controls, eight blank regions on the chip were separately collected, which then went through the same lysis and MDA steps. Seven of the eight samples turned negative in the following gel electrophoresis analysis, but a ~500 bp fragment from one sample was detected (Figure S1), which might be due to the nonspecific amplification from environmental DNA contamination or primer dimers when performing MDA^20^, ^21^. Based on our preliminary experiment and results (data not shown), the incidence of this nonspecific amplification was relatively low (1%–3%).

Out of the 60 MDA reactions, the target 16S ribosomal RNA (rRNA) gene fragments of 24 FADA‐labeled cells and 12 Cy5ADA‐labeled cells were amplified, respectively. These 16S rRNA gene amplicons were then analyzed by Sanger sequencing. As expected, sequences of FADA‐labeled cells were all identified as *E. coli* and Cy5ADA‐labeled cells were all identified as *B. subtilis*, except for four samples (two FADA‐labeled cells and two Cy5ADA‐labeled cells) that were discarded because they failed in the sequencing quality control. In the end, FLCiSS achieved 73.3% (22/30) and 33.3% (10/30) success rates in identifying *E. coli* and *B. subtilis* at the single‐cell level, respectively, which were comparable with some of the best ratios reported in recent single‐bacterium sequencing studies^7^, ^22^, ^23^. The higher success rates of Gram‐negative bacteria, a phenomenon commonly observed in single‐bacterium sequencing^24^, is probably because the thick cell wall of Gram‐positive bacteria was more difficult to lyse, especially when handled with single cells. Taking these data together, the feasibility of FLCiSS in integratively providing fluorescence labeling data and taxonomic identification of individual bacterium is proved.

### Single‐cell growth pattern analysis and taxon identification of gut microbiota

**Figure 3:**
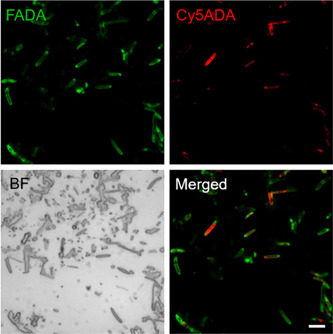
Confocal fluorescence imaging of the FDAA‐labeled gut microbiota using FLCiSS protocol. The cecal microbiota of mouse sequentially received FADA (green) and Cy5ADA (red) was collected and imaged. Scale bars = 2 μm.

After confirming the practicability of FLCiSS, we applied it to the analysis of mouse gut microbiota. Using a sequential in vivo labeling protocol that we previously developed^14^, ^17^, mouse gut microbiota that was labeled by two FDAA signals (containing the cell growth and division information) was obtained. The fluorescence labeling of the gut bacteria could be readily observed by confocal imaging (Figure 3). However, because of the requirement of subsequent cell sorting, coverslip and oil immersion lens could not be used here, which resulted in the reduced spatial resolution of the bacteria compared with our previous reports^14^, ^17^.

After optimization, 210 cells in total were imaged and sorted individually, based on their labeling patterns and cellular morphologies. Following cell lysis, their gDNA was amplified via MDA and 90 amplifications (42.8%) were successful. The 16S rDNA of these 90 samples was then cloned and 59 samples passed the quality control, which were then sequenced to identify the taxa of each sample. Via comparing with the NCBI public nucleotide database (https://blast.ncbi.nlm.nih.gov/Blast.cgi), 48 cells were identified. These cells were classified at either the order, family, genus, or species level, and 11 samples (18.6%) were noncategorizable, which were listed here as uncultured bacteria (Table 1). The relatively low taxa resolution in identification and the high ratio of noncategorizable cells indicate that a large portion of the mouse gut bacteria was still not yet well characterized^25^.

**Table 1:** Bacteria imaged and taxonomically identified by FLCiSS.

Name	Taxonomic level	Gram stain	Total No./Pct (%)	Representative image
*Anaerotignum*	Genus	+^a^	9/15.3	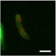
*Lachnospiraceae*	Family	/	8/13.6	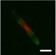
*Eubacteriales*	Order	/	4/6.8	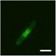
*Virgibacillus halodenitrificans*	Species	+	3/5.1	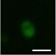
*Oscillibacter*	Genus	−^b^	2/3.4	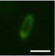
*Butyricicoccus*	Genus	+	2/3.4	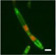
*Muribaculum*	Genus	−	2/3.4	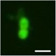
*Oscillospiraceae*	Family	/	2/3.4	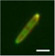
*Corynebacterium propinquum*	Species	+	1/1.7	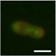
*Enterocloster clostridioformis*	Species	+	1/1.7	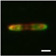
*Eisenbergiella*	Genus	+	1/1.7	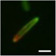
*Flavonifractor*	Genus	+	1/1.7	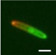
*Corynebacterium segmentosum*	Species	+	1/1.7	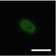
*Pseudoflavonifractor*	Genus	+	1/1.7	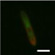
*Candidatus arthromitus*	Species	+	1/1.7	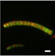
*Clostridium*	Genus	+	1/1.7	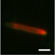
*Prevotellamassiliatimonensis*	Species	−	1/1.7	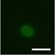
*Akkermansia muciniphila*	Species	−	1/1.7	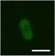
*Vampirovibrio chlorellavorus*	Species	−	1/1.7	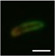
*Moraxella osloensis*	Species	−	1/1.7	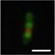
*Acinetobacter ursingii*	Species	−	1/1.7	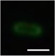
*Aquicella*	Genus	−	1/1.7	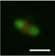
*Phyllobacteriaceae*	Family	/	1/1.7	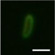
*Hyphomicrobiaceae*	Family	/	1/1.7	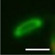
Uncultured bacterium		/	11/18.6	
				

^a^ + , Gram‐positive; ^b^−, Gram‐negative. Total No., the number of isolates successfully identified in each taxon; Pct, the percentage of this taxon in the total population identified. Scale bars, 2 μm.

A representative fluorescence image for each sample cluster showing their growth/division patterns is included in Table 1. Most Gram‐positive bacteria were either spindle‐shaped (*Butyricicoccus*, *Eisenbergiella*, *Clostridium, Enterocloster clostridioformis*), curved (*Anaerotignum*), straight (*Flavonifractor*), or small (*Virgibacillus halodenitrificans*, *Corynebacterium segmentosum*) rods. Of note, a *Corynebacterium propinquum* cell having a characteristic club shape was identified^26^. Many of the Gram‐positive bacteria, like *Anaerotignum*, *Lachnospiraceae*, *Butyricicoccus*, and *Enterocloster clostridioformis*, divided by binary fission with red‐labeled septum in the mid‐cell. In contrast, *Eisenbergiella* and *Flavonifractor* presented asymmetrical distribution of the red signal.

Most Gram‐negative bacteria shown here were either short (*Oscillibacter*, *Aquicella*, *Acinetobacter ursingii*, *Akkermansia muciniphila*) or oval (*Muribaculum*, *Prevotellamassilia timonensis*) rods. A slightly curved cell was identified as *Vampirovibrio chlorellavorus*, which was reported to be in coccus shape^27^, and this inconsistency could be explained by the pleomorphic nature of this species^28^. It is worth noting that a *Moraxella osloensis* cell presenting a characteristic diplobacilli morphology (brick shaped)^29^ was recognized. Owing to their thinner peptidoglycan, most of these Gram‐negative bacteria had relatively weak FDAA labeling, especially the Cy5ADA signal (the labeling time was shorter).

Fluorescence images of the rest of the cells that were successfully identified in sequencing are demonstrated in Figure S2. The cellular length, width, and FDAA‐labeling intensity information of the identified bacteria were listed in Table S1 (labeling intensities were categorized into four levels based on the fluorescence imaging results). Not surprisingly, the morphologies and labeling patterns of some of the cells belonging to the same order (*Eubacteriales*; Figure S2C) or family (*Lachnospiraceae*; Figure S2B) showed considerable heterogeneities. In comparison, the nine cells identified as the genus *Anaerotignum* (Figure S2A) had relatively uniform cellular shapes and labeling patterns. For cells identified at either genus or species level, the Gram‐positive and Gram‐negative bacterial isolates accounted for 69% and 31%, respectively. The higher percentage of Gram‐positive bacterial isolates was probably because of their stronger and clearer FDAA labeling, which made us more inclined to select them during imaging.

**Figure 4:**
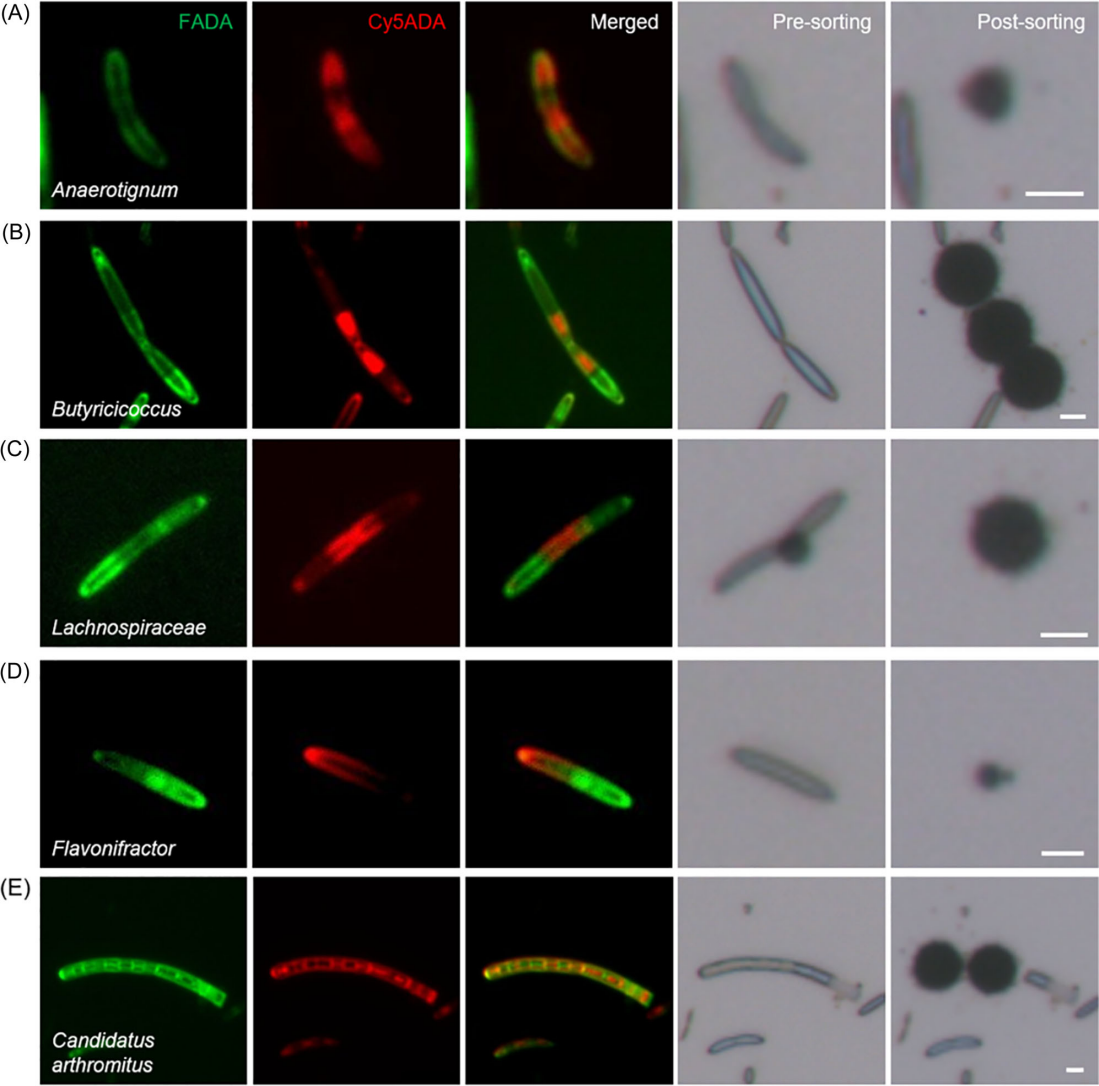
Representative fluorescence images of the two‐colored gut bacteria that were sorted and identified after sequencing, and the corresponding bright‐field views before and after cell sorting. Scale bars = 2 μm.

To take a close look at the fluorescently imaged and sorted bacteria, the two fluorescent channels and bright‐field views (both before and after sorting) of some successfully identified microbial cells are shown in Figure 4. The three cells belonging to the genera *Anaerotignum* and *Butyricicoccus* and family *Lachnospiraceae*, respectively, presented clear green signals on the peripheral and red signals in the mid‐cell (Figure 4A–C), indicating that they probably divided in binary fission. The fact that only one pole of the cells had strong red signals suggested that they might have already finished one cell division during the 6 h labeling period since the unipolar red‐labeling could be the remnants of the strongly labeled septum in the previous cell cycle^17^. A cell belonging to the genera *Flavonifractor* showed asymmetrical distribution of the two colors (Figure 4D). This may be because it was a daughter cell that just separated from the mother cell whose septum would have had strong red labeling; another explanation is that it is a type of bacteria that normally grows from one end. Of special note, cells with characteristic features of segmented filamentous bacteria (or *Candidatus arthromitus*)^30^ were also observed by fluorescence imaging in the sample (Figure 4E). Satisfyingly, consistent taxonomic identity was attained in 16S rDNA sequencing, which again showcased the reliability of our protocol. Taken these data together, this FLCiSS strategy demonstrated its potential as a powerful tool for identifying the taxonomy of gut bacterial cells that had informational fluorescence signals at a single‐cell resolution with a high throughput.

### Whole‐genome assembly of gut bacteria isolated by FLCiSS

Furthermore, to assess the quality of the single‐cell genomes obtained from FLCiSS, we selected 10 gDNA samples having positive 16S rRNA gene amplicons for library construction and whole‐genome sequencing. After genome assembly, four cells were successfully identified as *Flavonifractor plautii* (previously identified as genus *Flavonifractor*), *Muribaculum gordoncarteri* (previously identified as genus *Muribaculum*), *Akkermansia muciniphila*, and *Candidatus arthromitus*. The quality of these four single‐cell amplified genomes (SAGs) was also evaluated. The completeness of SAGs ranged from 45.3% to 97.8% (Table 2). Moreover, the SAGs of *Candidatus arthromitus* represented near‐complete genomes (97.8% completeness), and four SAGs presented nearly all rRNA and transfer RNA. These data verified the feasibility of FLCiSS in providing high coverage genomes from one gut bacterial cell with low contamination rates (1.3%–3.9%).

**Table 2:** Genome sequencing of gut bacterial cells via FLCiSS.

Species	Contigs	Largest contig (kb)	Total length (kb)	GC (%)	N50 (kb)	Completeness (%)	Contamination (%)	16S rRNA	23S rRNA	5S rRNA	tRNA
*Flavonifractor plautii*	694	53.2	2314	53.2	8.9	46.0	1.7	1	0	0	24
*Muribaculum gordoncarteri*	265	36.1	1104	49.7	13.2	45.3	1.9	3	2	3	27
*Akkermansia muciniphila*	348	71.8	1827	55.0	19.9	64.1	1.3	1	1	1	34
*Candidatus arthromitus*	363	153.4	2358	31.8	34.4	97.8	3.9	1	1	1	27

GC, genomic content; rRNA, ribosomal RNA; tRNA, transfer RNA.

In this study, our FLCiSS strategy allows linking the growth/division pattern data with the genomic information of the highly diverse gut microbiota on a single‐cell basis. Several factors that can further improve this technique should be considered. First, a more efficient cell lysis protocol, especially for Gram‐positive bacteria, will certainly increase the success rates in single‐bacterium analysis. Second, a better imaging setup (e.g., a more flattened microbial sample on the slide) will enable imaging of the fluorescently labeled bacteria with a higher spatial resolution. Third, as the gut microbiome database continues to expand, the identification rates and taxonomic resolution of the sequenced single bacterium will be improved. Last, FDAA probes with smaller molecule weights, such as hydroxycoumarin‐carbonyl‐amino‐d‐alanine and nitrobenzoxadiazole‐amino‐d‐alanine^15^, might improve the labeling efficiency of some Gram‐negative bacteria in the gut. In addition to the mammalian gut microbiota, this strategy should also be applicable to bacterial samples collected from a great variety of sources, for example, environmental microbiotas from the soil, the air, and the aquatic habitats. The integrative use of confocal fluorescence microscopy with single‐cell sequencing may help advance our understanding of these complex ecosystems. Moreover, besides FDAA labeling, other fluorescent‐tagging methods that are suitable for bacterial imaging and capable of providing phenotypic information, green fluorescent protein, bio‐orthogonal metabolic labeling probes^31^, fluorescent antibodies, to name a few, should also be compatible with the single‐cell sorting and sequencing protocol proposed herein. It is reasonable to believe that this FLCiSS strategy has great potential to achieve the aim of “what you see is what you get” in microbiology.

## MATERIALS AND METHODS

### Bacterial strains and growth conditions

*E. coli K12* (HfrH) and *B. subtilis* (CICC 23659) were purchased from the China Center of Industrial Culture Collection. They were first inoculated on Luria–Bertani (LB) agar plates overnight at 37°C, and the fresh bacterial clones were inoculated into the LB broth medium for routine culture.

### Reagents

FDAA probes were purchased from the Chinese Peptide Company. The REPLI‐g Mini Kit was purchased from Qiagen. Lysis buffer for direct PCR of microorganisms was purchased from Takara Biomedical Technology. The 2× *Taq* PCR mix, RNase‐free ddH_2_O, and phosphate‐buffered saline (PBS) were purchased from Sangon Biotech.

### Animals

Male 8‐week‐old C57BL/6‐specific pathogen‐free mice were purchased from Huafukang Biotechnology. Mice were bred in the animal facility of Renji Hospital in a temperature‐controlled environment (25°C) with a 12‐h light/dark cycle and received a standard chow diet and clean water. All animal experiments were performed in accordance with guidelines approved by the Institutional Animal Care and Use Committee of the Shanghai Jiao Tong University School of Medicine.

### Procedure

#### Bacterial strains labeling with FDAA probes

*E. coli* and *B. subtilis* strains were cultured in LB medium at 37°C till the mid‐exponential phase. FADA or Cy5ADA (at a final concentration of 0.3 mM) was added to the *E. coli* and *B. subtilis* cultures, respectively. After labeled for 3 h, the bacterial suspensions were washed with sterile PBS (2 × 1 ml) and ddH_2_O (2 × 1 ml). Then, equal volumes of *E. coli* and *B. subtilis* suspensions labeled with FDAA probes were mixed together and diluted with ddH_2_O for further analysis.

#### Sequential labeling of gut microbiota in vivo with FDAA probes

The C57BL/6 mice were sequentially administered with 200 μl of FADA and Cy5ADA (1 mM in ddH_2_O) by oral gavage with an interval of 3 h. After sequential labeling, their cecal microbiotas were collected based on a previously reported protocol^14^. In brief, the cecum was dissected and minced with a pair of iris scissors in 2 ml of PBS. Then, the tissues were filtered with a 40 μm sterile cell strainer to remove nonbacterial materials. The filtrates were then centrifuged and washed with PBS (2 × 1 ml) and ddH_2_O (2 × 1 ml) at 15,000 *g*, 3 min. The cecal microbiota was then resuspended in ddH_2_O for subsequent analysis.

#### Confocal fluorescence microscopy and cell sorting

Freshly labeled bacteria (10 μl) were loaded onto a HOOKE HSC24 chip (HOOKE Instruments Ltd.) without a cover slide for imaging and sorting. The chip was divided into four quadrants with *X*‐ and *Y*‐axis to locate targeted cells. The slide was then air‐dried for 10–20 min in a fume hood. Confocal fluorescence imaging was performed on a HOOKE S3000 spinning disk confocal microscope equipped with a ×100 air‐immersion objective lens (HOOKE Instruments Ltd.). Samples were excited at 488 nm for FAM and 639 nm for Cy5, and the emission was detected using corresponding emission filters. After imaging, the chip was transferred to a laser ejection equipment (PRECI SCS; HOOKE Instruments Ltd.), and the targeted single cell was sorted into an HSR04 receiver (HOOKE Instruments Ltd.) via laser ejection technology.

#### MDA of one bacterial cell

The single‐cell receiving tube contained 3 μl of buffer D2 prepared according to the REPLI‐g Mini Kit instruction (Qiagen) and 1 μl of PCR lysis buffer (Takara). After sorted, bacterial cells were ruptured immediately with three freeze–thaw cycles using liquid nitrogen. Then, the sample was incubated at 65°C for 10 min, after which 0.6 μl of neutralization buffer (supplied in the REPLI‐g Mini Kit) was added. The Phi29 DNA polymerase buffer (Qiagen) prepared on ice was then added to the reaction to amplify the gDNA released from the lysed single cells. After amplification at 30°C for 10 h, the Phi29 DNA polymerase was denatured at 65°C for 5 min. The obtained amplification products were purified using a Genomic DNA Kit (Qiagen). Subsequently, the quality and quantity of each DNA solution were checked by NanoDrop 2000 spectrophotometer (Thermo Fisher Scientific). The gDNA products of a single bacterial cell were stored at −80°C before further sequencing and analysis.

#### 16S rRNA gene amplification and sequencing

The full length of the bacterial 16S rRNA gene was amplified using the primers 27F (5′‐AGAGTTTGATCCTGGCTCAG‐3′) and 1492R (5′‐TACGGYTACCTTGTTACGACTT‐3′) at an annealing temperature of 51°C^32^. The PCR mixtures contained 12.5 μl 2× *Taq* PCR Mix (Sangon), 0.2 μM forward primers, 0.2 μM reverse primers, and 10 ng template, brought up to a final volume of 25 μl with ddH_2_O. The amplification products were visualized by electrophoresis on 1.0% agarose gels and purified with the Gel Advance gel extraction system (Viogene). Then, the purified products were cloned into the pUCmT Vector and transformed into *E. coli* XL1‐Blue for growth. Clones were randomly screened from each single‐cell sample and sequenced using an ABI Prism 3100 genetic analyzer system (Applied Biosystems).

The quality of each sequence was checked by the Phred/Phrap program^33^. High‐quality sequences were compared with the NCBI public nucleotide database using BblastN software (https://blast.ncbi.nlm.nih.gov/Blast.cgi) to obtain the approximate taxonomic identification of the single bacterium. The sequences have been submitted in GenBank, with accession numbers OP090257–OP090315.

#### Whole‐genome sequencing of a single bacterial cell

According to the 16S rRNA gene sequencing results, the samples yielding positive 16S rRNA gene amplicons were selected and then library construction was performed for shotgun sequencing using the Illumina Novaseq. 6000 platform (Novogene Co., Ltd.). Raw data were trimmed using fastp 0.21.0^34^. Genome assembly was carried out via SPAdes v3.15.0 (‐sc ‐careful)^35^ and quality was assessed via Quast^36^. CheckM v1.0.13^37^ was used to estimate the completeness of the genome. Prokka was used to analyze additional quality assurance metrics, including the annotation of noncoding RNAs^38^. All the de novo assembled genomes are deposited on NCBI under SRA accession numbers SAMN30089416–SAMN30089419.

## AUTHOR CONTRIBUTIONS

Juan Gao, Chaoyong Yang and Wei Wang designed the study. Juan Gao and Di Sun performed experiments and analyzed the data. Bei Li contributed to designing the analytical protocols. Juan Gao and Wei Wang wrote the manuscript. Chaoyong Yang and Wei Wang supervised the project.

## ETHICS STATEMENT

All animal experiments were performed in accordance with the guideline approved by the Institutional Animal Care and Use Committee of the Shanghai Jiao Tong University School of Medicine.

## CONFLICT OF INTERESTS

The authors declare no conflict of interests.

## Supporting information

Supporting information.

## Data Availability

Data supporting this study are available within the paper and its Supporting Information files.
