# Supplementary material for: Integrated identification of growth pattern and taxon of bacterium in gut microbiota via confocal fluorescence imaging‐oriented single‐cell sequencing
Source: mLife. 2022 Sep 26;1(3):350–8. doi: 10.1002/mlf2.12041 (PMC10989894; doi:10.1002/mlf2.12041)
Supplement: Supplementary file 1 — Supporting information. [file MLF2-1-350-s001.pdf]

Integrated Identification of Growth Pattern and Taxon of Bacterium in Gut Microbiota via Confocal Fluorescence Imaging-oriented Single-cell Sequencing

Juan Gao[1,#], Di Sun[1,#], Bei Li[2], Chaoyong Yang[1,3]*, Wei Wang[1,*]

[1]Institute of Molecular Medicine, Shanghai Key Laboratory for Nucleic Acid Chemistry and Nanomedicine, Renji Hospital, Shanghai Jiao Tong University School of Medicine, Shanghai, 200127, China

[2]State Key Laboratory of Applied Optics, Changchun Institute of Optics, Fine Mechanics and Physics, Chinese Academy of Sciences, Changchun, 130033, China

[3]The MOE Key Laboratory of Spectrochemical Analysis and Instrumentation, Key Laboratory for Chemical Biology of Fujian Province State Key Laboratory of Physical Chemistry of Solid Surfaces, Department of Chemical Biology, College of Chemistry and Chemical Engineering, Xiamen University, Xiamen 361005, China

Supporting Information

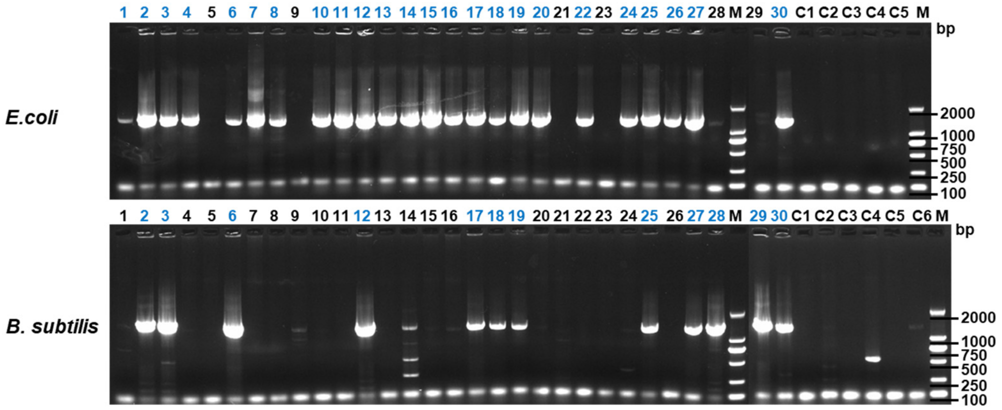

Figure S1. Amplified 16S rRNA gene region of the cells sorted from a mock microbial community resolved by agarose gel electrophoresis. The 16S rRNA gene fragments of single *E. coli* or *B. subtills* cells were amplified from their MDA products and detected by agarose gel electrophoresis. Positive *E. coli* and *B. subtills* samples were sequenced and shown in blue font. 1-30, single-cell samples; C1-C4, negative controls of MDA; C5-C6 negative controls of 16S rRNA gene amplification. Lanes M, molecular size markers.

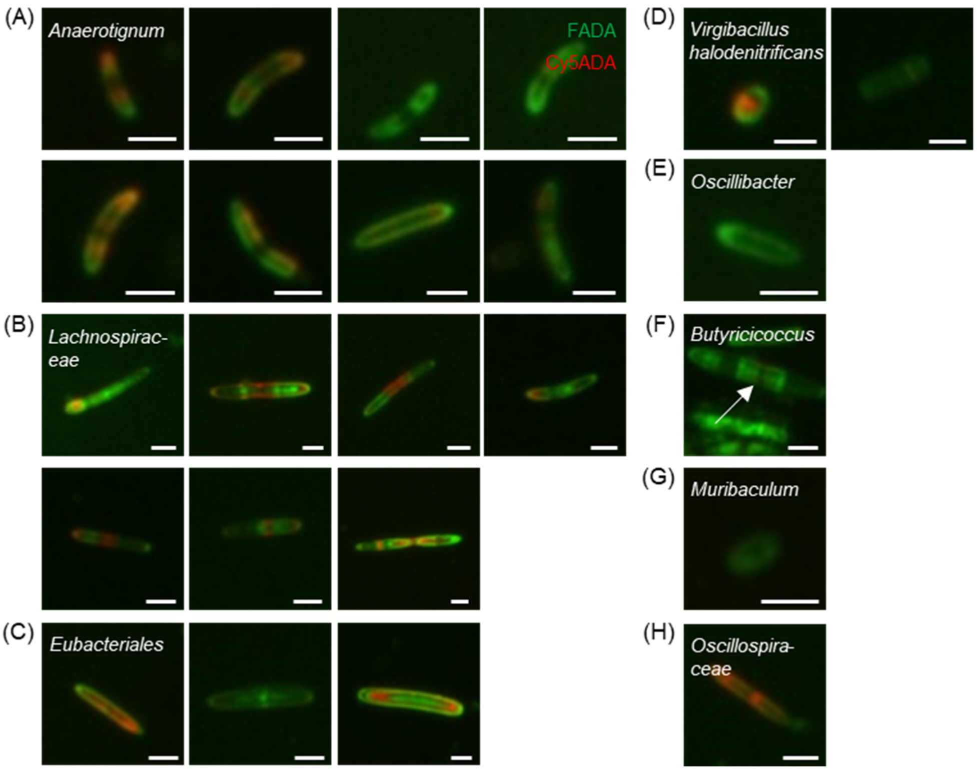

Figure S2. Fluorescence images of the cells taxonomically identified by FLCiSS. Scale bars, 2 μm.

Table S1 Cell shape and labeling intensity information of different taxa.

Taxon name	Length (μm)	Width (μm)	Fluorescence intensity [c]
Anaerotignum	4.41[a] ± 0.91[b]	0.85±0.14	++
Lachnospiraceae	8.09 ± 2.56	1.17±0.29	++
Eubacteriales	7.41 ± 2.39	1.43±0.48	+++
Virgibacillus halodenitrificans	2.60 ± 1.16	1.20±0.38	+
Oscillibacter	2.74 ± 0.45	0.94±0.14	+
Butyricicoccus	13.51 ± 4.43	1.65±0.09	+++
Muribaculum	3.47 ± 0.38	1.43±0.38	++++
Oscillospiraceae	5.94 ± 0.10	1.05±0.30	++
Corynebacterium propinquum	3.80	1.43	+
Enterocloster clostridioformis	8.80	1.35	+++
Eisenbergiella	5.91	1.31	+++
Flavonifractor	6.24	1.16	+++
Corynebacterium segmentosum	1.96	1.20	+
Pseudoflavonifractor	6.47	0.73	+
Candidatus arthromitus	20.31	1.79	+++
Clostridium	7.78	1.10	+++
Prevotellamassilia timonensis	1.89	1.26	+
Akkermansia muciniphila	2.18	0.96	+
Vampirovibrio chlorellavorus	4.05	1.07	+
Moraxella osloensis	4.03	0.98	+
Acinetobacter ursingii	2.62	0.91	+
Aquicella	2.47	0.95	+
Phyllobacteriaceae	2.81	0.93	+
Hyphomicrobiaceae	2.64	0.84	++

[a], mean of the cell length/width of each taxon; [b], SD values of the cell length/width of each taxon; [c], the fluorescence intensities of labeled cell are categorized into four levels (+ to ++++, weakest to strongest).
